# Genome-Wide Mining of *Selaginella moellendorffii* for Hevein-like Lectins and Their Potential Molecular Mimicry with SARS-CoV-2 Spike Glycoprotein

**DOI:** 10.3390/cimb45070372

**Published:** 2023-07-14

**Authors:** Ahmed Alsolami, Amina I. Dirar, Emadeldin Hassan E. Konozy, Makarim El-Fadil M. Osman, Mohanad A. Ibrahim, Khalid Farhan Alshammari, Fawwaz Alshammari, Meshari Alazmi, Kamaleldin B. Said

**Affiliations:** 1 Department of Internal Medicine, College of Medicine, University of Ha’il, Ha’il 55476, Saudi Arabiakf.alshammari@liveuohedu.onmicrosoft.sa (K.F.A.); 2 Medicinal, Aromatic Plants and Traditional Medicine Research Institute (MAPTRI), National Center for Research, Mek Nimr Street, Khartoum 11111, Sudan; 3 Department of Biotechnology, Africa City of Technology (ACT), Khartoum 11111, Sudan; makarim84@act.gov.sd; 4 Pharmaceutical Research and Development Centre, Faculty of Pharmacy, Karary University, Omdurman, Khartoum 11111, Sudan; 5 Department of Data Science, King Abdullah International Medical Research Center (KAIMRC), Riyadh 12211, Saudi Arabia; 6 Department of Dermatology, College of Medicine, University of Ha’il, Ha’il 55476, Saudi Arabia; fawwazf@liveuohedu.onmicrosoft.sa; 7 College of Computer Science and Engineering, University of Ha’il, Ha’il 81451, Saudi Arabia; 8 Department of Pathology and Microbiology, College of Medicine, University of Ha’il, Ha’il 55476, Saudi Arabia; kbs.mohamed@uoh.edu.sa; 9 Genomics, Bioinformatics and Systems Biology, Carleton University, 1125 Colonel-By Drive, Ottawa, ON K1S 5B6, Canada

**Keywords:** Hevein-lectins, *Selaginella moellendorffii*, SARS-CoV-2, spike protein, docking, variants

## Abstract

Multidisciplinary research efforts on potential COVID-19 vaccine and therapeutic candidates have increased since the pandemic outbreak of SARS-CoV-2 in 2019. This search has become imperative due to the increasing emergences and limited widely available medicines. The presence of bioactive anti-SARS-CoV-2 molecules was examined from various plant sources. Among them is a group of proteins called lectins that can bind carbohydrate moieties. In this article, we present ten novel, chitin-specific Hevein-like lectins that were derived from *Selaginella moellendorffii* v1.0’s genome. The capacity of these lectin homologs to bind with the spike protein of SARS-CoV-2 was examined. Using the HDOCK server, 3D-modeled Hevein-domains were docked to the spike protein’s receptor binding domain (RBD). The Smo446851, Smo125663, and Smo99732 interacted with Asn343-located complex *N*-glycan and RBD residues, respectively, with binding free energies of −17.5, −13.0, and −26.5 Kcal/mol. The molecular dynamics simulation using Desmond and the normal-state analyses via torsional coordinate association for the Smo99732-RBD complex using iMODS is characterized by overall higher stability and minimum deformity than the other lectin complexes. The three lectins interacting with carbohydrates were docked against five individual mutations that frequently occur in major SARS-CoV-2 variants. These were in the spike protein’s receptor-binding motif (RBM), while Smo125663 and Smo99732 only interacted with the spike glycoprotein in a protein–protein manner. The precursors for the Hevein-like homologs underwent additional characterization, and their expressional profile in different tissues was studied. These in silico findings offered potential lectin candidates targeting key *N*-glycan sites crucial to the virus’s virulence and infection.

## 1. Introduction

From the beginning of the coronavirus disease 2019 (COVID-19) caused by the pandemic outbreak of Severe Acute Respiratory Syndrome Coronavirus 2 (SARS-CoV-2) in Wuhan, China, late 2019, reports on the severe gaps in vaccine and therapeutics have come in from all over the world. With about 652 million confirmed cases and more than six million documented deaths, the disease’s worldwide mortality and morbidity are still rising [1]. The evolutionary dynamics of SARS-CoV-2 epidemics vary worldwide and depend on a range of variables, including host genetics, virus genetics, co-infection, environmental factors, country health infrastructures, and public attitudes and preventative strategies [2,3,4,5,6]. However, drug discovery and immunization programs at the international level continue to take precedence to control and prevent the disease. The SARS-CoV-2 is a large, positive-sense, single-stranded, RNA coronavirus. The virus produces four major structural proteins: spike (S), nucleocapsid (N), envelope (E), and membrane (M). The spike protein mediates viral infection of cells via the transmembrane zinc protein angiotensin-converting enzyme 2 (ACE2) receptor. Structurally, the S protein is a type-I membrane trimer protein constructed from a homodimer of two subunits (S1 and S2) linked by a third membrane-embedded serine 2 protease subunit [7]. The receptor-binding domain (RBD) is located at the top of the S1 subunit. The spike protein is highly glycosylated (*O*- and *N*-glycosylation); each monomer contains 22 *N*-glycosylation points, partially facilitating viral virulence. Furthermore, the *N*-glycans at the protein’s surface can mediate RBD protection against neutralization antibodies [8]. The *N*-glycan structures are synthesized from glycan core Glc3Man9GlcNAc2 precursor, which is then processed into three types, oligo-mannose (2HexNAc), hybrid (3HexNAc), and complex (<3HexNAc) structures [9]. Many drugs have been tested for their ability to inhibit the spike protein–angiotensin-converting enzyme-2 (ACE-2) interactions; they exert their potency by either blocking the RBD of the S protein [10] or obstructing the ACE2 binding domain [11]. Thus, despite enormous efforts being made for suitable candidates, there is yet a significant gap remaining to be closed in this area. While many products were developed, they were limited by global availability and/or other pitfalls in design. 

Lectins are carbohydrate-binding proteins with high specificity and avidity that bind carbohydrate moieties reversibly without changing their chemical structures [12]. Over the years, plant lectins have been extensively studied for their application as antimicrobial, anticancer, anti-inflammatory, antinociceptive agents, etc. [13,14,15]. Several plant lectins have been identified as SARS-CoV-2 inhibitors. They are thought to bind the *N*-glycan structure near the RBD, thereby preventing the S protein from binding the ACE2. These interactions may also result in conformational adjustments that facilitate antibodies’ access to the target’s concealed epitopes and lead to immunological neutralization [14,16,17]. Man-specific lectins belonging to the families’ legume (i.e., ConA *Canavalia ensiformis* and LcA *Lens culinaris*), Jacalin (i.e., BanLec *Musa acuminata*), Nictaba (i.e., Nictaba *Nicotiana tabacum*), Ricin- B (i.e., IraB *Iris hollandica*), and GNA (i.e., Gastrodianin *Gastrodia elata*) were reported as coronaviruses’ spike protein blockers that interact with the oligo-mannose structures located near the RBD [18]. Instead of the typical carbohydrate–protein interactions, lectins can also engage in protein–protein interactions with the spike protein. A chitin-specific lectin *Urtica dioica* Agglutinin (UDA) isolated from the rhizomes of the *Urtica dioica* inhibits SARS-CoV-2 infection by binding to the RBD of the spike protein in a protein–protein manner [19]. 

In this in silico study, and in order to discover an inhibitory protein candidate for the SARS-CoV-2 spike protein, we analyzed members of the Hevein-like lectins (chitin-specific lectins) found in the genome of the spike moss plant (*Selaginella moellendorffii*, Family: Selaginellaceae), for the possibility of interacting with the spike protein’s *N*-glycans and functioning as potential SARS-CoV-2 inhibitors. To acquire a better understanding of the physical underpinnings of the complexes, the modeled proteins were docked against the RBD of the spike protein. The complexes were then further examined using molecular dynamics simulation on Desmond and the iMODS server to provide an overview of the physical bases of the complexes. Because of its bioactive metabolites (primary and secondary), which are employed in both conventional and modern medicine as antifungal, antibacterial, antiviral, anticancer, anti-inflammatory, anti-allergy, and antioxidant agents, the spike moss was selected as a prospective source for lectin [20]. A comprehensive overview of the domain architecture, gene architectures, genomic expansion, evolutionary relationship, and expressional profiles in various tissues and organs will also be provided by the study.

## 2. Materials and Methods

### 2.1. Screening the Spike Moss Genome for Putative Hevein-like Lectin Genes

The genome assembly (scaffold level) of *Selaginella moellendorffii* (v1.0, ID:88036) [21] found in Phytozome v13 (the plant genomics resources) (available at https://phytozome-next.jgi.doe.gov/, accessed 14 December 2022) [22] was searched for Hevein-like lectin genes by aligning the proteome against the sequence of *Hevea brasiliensis* agglutinin (Hevein–GenBank: ABW34946.1, Pfam: PF00187) using Phytozome-BLASTP tool (E value < 0.0001, Matrix: BLOSUM62). The sequence with the highest identity was used for a second BLAST search. The candidate Hevein-like lectin sequences obtained was then retrieved using the Phytozome-BioMart tool, and each sequence was checked for the presence of at least one Hevein-like domain using the InterProScan 5 [23] (available at (http://www.ebi.ac.uk/interpro/), accessed 15 December 2022). The spike moss Hevein-like putative genes structure was studied by investigating the exon/intron organization of each gene coding sequence (CDS) in reference to their genomic DNA sequences using the Gene Structure Display Server (GSDS) (available at (http://gsds.gao-lab.org/), accessed 15 December 2022) [24]. 

### 2.2. Analysis of Hevein-like Gene Expansion and Evolutionary Relationship

The Hevein-like putative lectin gene IDs were checked against the Pant Duplicate Gene Database PlantDGD (available at (http://pdgd.njau.edu.cn:8080/), accessed 19 December 2022) [25] to identify the type of duplication and the duplicate genes, then the synonymous substitution (Ks) was calculated using the Ka/Ks calculator found in the TBtools v1.0986853 [26]; values higher than 1 were excluded. The phylogenetic trees were constructed using aligned trimmed lectin-like domain sequences. The maximum likelihood method and the JTT matrix-based substitution model were used in MEGA X to analyze the evolutionary relationship [27]. The final bootstrap for consensus trees was inferred from 1000 replicates.

### 2.3. Expressional Profile of Hevein-like Genes Based on Publicly Available Resources

The dataset of expression profiling of Selaginella moellendorffii analyzed by high throughput sequencing and deposited in the NCBI Gene Expression Omnibus (GEO) (GSE123120) was downloaded [28]. The GSE123120 file containing RNA-seq expression raw read counts per gene for semplice replicates (seeds, rhizophore, leaf, and shoot) was selected for Hevein-like genes read counts. Data were normalized, and the differential expression was analyzed by the Integrated Differential Expression and Pathway analysis (available at (http://bioinformatics.sdstate.edu/idep96/) accessed 15 April 2023) (iDEP, v0.96 [29]. Each Hevein-like coding sequence (CDS) was searched for miRNA targeting sites using the Plant Small RNA Target Analysis Server (psRNATarget, v2) under default setting (available at (https://www.zhaolab.org/psRNATarget/analysis?function=1), accessed 23 January 2023) [30]. 

### 2.4. Characterization of Hevein-like Homologs 

The protein sequences of Hevein-like lectin homologs were checked for the presence of signal peptide and transmembrane domain sequences using the SignalP 5.0 server [31] (available at (https://services.healthtech.dtu.dk/service.php?SignalP-5.0), accessed 19 December 2022) and TMHMM 2.0 [32] (available at (https://services.healthtech.dtu.dk/service.php?TMHMM-2.0), accessed 19 December 2022), respectively. Furthermore, each sequence was analyzed for the presence of potential glycosylation sites for *N*-glycan using NetNGlyc 1.0 server [33] (available at (https://services.healthtech.dtu.dk/service.php?NetNGlyc-1.0), accessed 19 December 2022) and *O*-glycan using NetOGlyc 4.0 server (available at (https://services.healthtech.dtu.dk/service.php?NetOGlyc-4.0), accessed 19 December 2022) [34]. The subcellular localization was predicted using the WoLF PSORT webserver [35] (available at (https://wolfpsort.hgc.jp/), accessed 19 December 2022), followed by the prediction of signal classes (signal peptide (SP), transmembrane (TM), intracellular (IC), and unconventional secretion (USP)) using the OutCyte 1.0 server (available at (http://www.outcyte.com/), accessed 19 December 2022) [36].

### 2.5. Secondary Structure Prediction, Structural Modeling, and Validation of Hevein-like Homologs

The secondary structures of the 10 Hevein-like sequences were determined using the SOPMA (available at (https://npsa-prabi.ibcp.fr/cgi-bin/npsa_automat.pl?page=/NPSA/npsa_sopma.html), accessed 8 January 2023) [37]. The 3D protein structures of the lectin sequences were built using the transform-restrained Rosetta (trRosetta) webserver (available at (https://yanglab.nankai.edu.cn/trRosetta/), accessed 8 January 2023) [38]. The structures obtained by trRosetta were validated by examining the Phi/Psi Ramachandran plot using SAVES v6.0 Structure Validation Server (available at (https://saves.mbi.ucla.edu/), accessed 8 January 2023) [39] acquired from the PROCHECK server [40], which evaluates the stereo-chemical properties of the modeled protein structures of lectins. Additionally, the PROSA (Protein Structure Analysis) webserver (available at (https://prosa.services.came.sbg.ac.at/prosa.php), accessed 14 January 2023) [41] was employed to examine the energy specifications of the protein models compared to the database structures. 

### 2.6. Retrieval and Pzre-Processing of SARS-CoV-2 Spike Glycoprotein and the Lectin Structures

The 3-dimensional (3D) crystal structure of the SARS-CoV-2 S glycoprotein, at a 2.80 Å resolution (total structure weight 438.26 KDa), was downloaded from the PDB databank [42] with PDB ID: 6VXX [43]. The Schrödinger suite’s Protein Preparation Wizard tool of Maestro software (Schrödinger Release 2021-2: Maestro) was used to pre-process and prepare the structure of the SARS-CoV-2 spike glycoprotein (PDB: 6VXX, designated as chain B) and the 10 Hevein-like structures. In this process, bond order was assigned, hydrogens were added, missing loops and side chains were fixed, and the uncapped N-termini and C-termini were capped with ACE (*N*-acetyl) and NMA (*N*-methyl amide) groups, respectively. The sugar cofactor, *N*-acetylglucosamine (NAG), was retained in the protein structure. Afterward, the protein was optimized using PROPKA at a pH of 7.4 and minimized energy using the OPLS4 force field [44]. 

### 2.7. Identifying the Binding Sites of the Spike Glycoprotein and Lectins for Macromolecular and Ligand Docking

To identify the structural details of SARS-CoV-2 S glycoprotein and the 10 Hevein-like lectins, the NCBI Conserved Domain Search was used (available at (https://www.ncbi.nlm.nih.gov/Structure/cdd/wrpsb.cgi), accessed 18 January 2023) [45].

### 2.8. In Silico Molecular Docking

The HDOCK server (available at (http://hdock.phys.hust.edu.cn/), accessed 15 April 2023) [46] was used for in silico macromolecular docking of the lectins to *N*-linked glycans of SARS-CoV-2 spike glycoprotein using a hybrid algorithm approach. The HDOCK server uses an FFT-based method to perform global docking using the input receptor and ligand structure to sample acceptable binding conformations. Furthermore, the HDOCK docking tool performs rigid body docking by considering both the protein and the ligand (protein) onto grids. The binding energy of macromolecules was used to evaluate their binding mode and rank them based on their docking energies. In the selection process, lectin–RBD complexes were selected based on two factors: the docking lowest energy and, second, the interaction with *N*-acetylglucosamine (NAG) that contributed to the best complex selection when using the PDBsum: Structural summaries of PDB entries webserver (available at (http://www.ebi.ac.uk/pdbsum), accessed 15 April 2023) [47]. The HawkDock server ((http://cadd.zju.edu.cn/hawkdock/), accessed 15 April 2023) [48] was used to calculate the MM/GBSA (Molecular Mechanics–Generalized Born Surface Area) free binding energy for the docked complexes of lectins with SARS-CoV-2 S glycoprotein. The MM/GBSA was calculated based on the ff02 force field, the implicit solvent model, and the GBOBC1 model (interior dielectric constant = 1). The system was minimized for 5000 steps with a cut-off distance of 12 Å for van der Waals interactions (2000 cycles of steepest descent and 3000 cycles of conjugate gradient minimizations) and expressed in kcal mol-1. The docking modes generated were analyzed and visualized using Discovery Studio 2021 [49], which were subsequently exported as an image. 

Docked lectins having intriguing interactions with the spike’s RBD were subjected to further molecular docking studies against Angiotensin-converting enzyme 2 (ACE2) to evaluate their interactions at the RBD–ACE2 interface. The molecular structure of ACE2 was first retrieved from the protein complex PDB ID 6LZG (resolution 2.5) [50] and pre-processed using Maestro Software’s Protein Preparation Wizard tool before being subjected to docking simulations. Protein–protein interactions (PPI) between the SARS-CoV-2 spike glycoprotein (PDB ID 6VXX) and ACE2 were investigated. This was followed by additional multiple-chain docking simulations between the RBD–lectin complex and ACE2 were performed utilizing the HDOCK server [46]. The High Ambiguity Driven Protein–protein Docking (HADDOCK) 2.4 tool was then used to perform simultaneous docking of multi-body complexes between spike’s RBD, lectin, and ACE2 [51]. The inputs’ 3D structures were uploaded to the HADDOCK 2.4 server. The protein structural calculations were guided by defining active residues based on the previous reports for 6VXX RBD [43], Smo-lectins (Smo446851, Smo125663, and Smo99732), and ACE2 [50]. The standard HADDOCK docking protocol consists of three stages: it0, it1, and itw (rigid-body docking, semiflexible refinement in torsion-angle space, and a final explicit solvent refinement, in that order). The produced models were refined using one of three methods: energy reduction (the default in HADDOCK2.4), clustering, and ranking the HADDOCK score. The BIOVIA Discovery Studio software release 2021 [49] was used to select and analyze the 3D docked models.

### 2.9. In Silico Mutant Spike Protein Interactions

Five mutation points (i.e., Lys417Asn, Leu452Arg, Thr478Lys, Glu484Lys, and Asn501Tyr) are reported in spike protein’s ACE 2-receptor binding motif (RBM) from many SARS-CoV-2 variants and were affected in the parental structure (PDB 6VXX) separately. The UCSF-chimera (v. 1.16) [52] command line was used to create the mutations. The mutated protein structures were then subjected to optimization and energy minimization using the protein preparation wizard tool of Maestro software (Schrödinger Release 2021–2022: Maestro). Five mutant structures were obtained, and each was docked against Smo446851, Smo125663, and Smo99732. The MM/GBSA free binding energy for each complex was calculated. Different combinations of these mutations also occur within the RBM from the variants (alpha, beta, gamma, delta, and omicron), were also used to modify the spike protein parental structure, and were docked with the mentioned lectins.

### 2.10. Hotspot Analysis of the SARS-CoV-2 RBD–Lectin Complex

The energetically significant hotspot residues at the interface region of SARS-CoV-2 S glycoprotein and lectins were identified using the KFC Server (Knowledge-based FADE and Contacts) (available at (https://mitchell-web.ornl.gov/KFC_Server/index.php), accessed on 27 February 2023) [53]. 

### 2.11. Normal-State Analyses via Torsional Coordinate-Association

The iMODS online server (available at (http://www.imods.chaconlab.org/), accessed on 24 March 2023) [54] was used to investigate the collective flexibility/motion functions of the lectin–spike glycoprotein complex based on normal-state analyses of their respective internal dihedral angle (torsional) coordinates [55]. Within the PDB file of lectin–spike glycoprotein docked complexes, the atoms/residues were continuously indexed. The complexes were uploaded to iMODS online server, parameters were kept as default, and the collective motions of proteins were investigated using normal mode analysis.

### 2.12. Molecular Dynamics Simulation (MDS)

The molecular dynamics simulation (MDS) of the docked spike’s RBD with potential lectins (Smo446851, Smo125663, and Smo99732) has been performed to analyze the complex stability in the physiological environment at (100 ns) using Desmond, a package of Schrödinger Release 2021–2022 [56]. The complexes were pre-processed using Protein Preparation Wizard of Maestro, which also included optimization and energy minimization of the complexes. The systems were subjected to solvation and ionization using the System Builder tool and solvated using a TIP3P (Transferable Intermolecular Interaction Potential 3 Points) water model in a 10 Å orthorhombic box. The models were made neutral by adding counter ions where needed. To mimic the physiological conditions, 0.15 M salt (NaCl) was added. The NPT ensemble (isothermal–isobaric: moles (N), pressure (P), and temperature (T) with 300 K temperature and 1 atmospheric pressure was selected for complete simulation. The models were relaxed before the simulation. Energy minimization of the system was performed using the steepest descent method, and the OPLS_2005 force field was used in the simulation [57]. The complex’s stability was assessed using 1000 frames generated from 100 ns MD simulation trajectory data. The trajectories were analyzed by plotting root mean square deviation (RMSD), root mean square fluctuation (RMSF) of the α-carbons of systems during the simulations, a radius of gyration (*R*g), solvent-accessible surface area (SASA), and intermolecular hydrogen bond profile of active site of complexes.

## 3. Results

### 3.1. General Overview of Hevein-like Lectins

**Figure 1:**
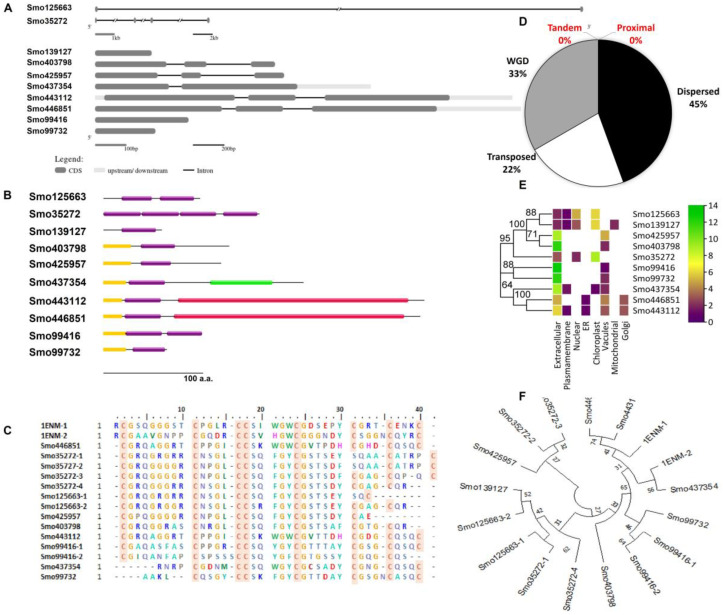
Characterization of Hevein-like homologs from *Selaginella moellendorffii*. (**A**) Gene structure and (**B**) domain architecture (Hevein domains = purple boxes (PF00187), red domain = chitinase class I (PF00182), green domain = lytic transglycosylase domain (PF03330), and yellow domain = signal peptide). (**C**) Multiple sequence alignment of trimmed Hevein domains (red box = conserved cysteine residue) and (**D**) the duplication of the Hevein genes and the mechanism type. (**E**) Predicted subcellular localization of Hevein-like homologs and (**F**) phylogenetic tree of trimmed Hevein domains and their evolutionary relationship (bootstrap 1000, the lectin ID and accession numbers are followed by the number of the domains which reside in the same protein and indicated after the dash). Urtica dioica agglutinin (1ENM) was used as a reference for alignment and phylogenetic analysis.

Searching the Selaginella moellendorffii (v1.0, ID:88036) genome for Hevein-like homologs resulted in identifying 10 sequences located in different scaffolds. A total of 30% of the identified lectins were secreted *O*-glycosylated chimerolectins. In these, the Hevein domain fused by the C-terminal to a chitinase class I domain (PF00182) (Smo443112 and Smo446851, ~34 KDa) translated from three exons in each gene or to a lytic transglycosylase domain (PF03330) as in the 20.9 KDa sequence Smo437354. Merolectins (40%) and Hololectins (30%) were also reported. For instance, the Smo425957 duplicated by a transposition mechanism from the Smo403798 were both Merolectins predicted as extracellular proteins. These were attached by the N-terminal to a signal peptide and by the C-terminal to a stretch of the non-conserved region of <50 amino acids in length. Their respective genes were constructed from three exons interrupted by two introns. Two and four tandemly arrayed Hevein-like domains shared an evolutionary relationship with other Merolectins, namely Smo139127, Smo425957, and Smo99732. Domains from Smo35272 4-domain Hololectin were believed to be expanded from Smo139127 by the mechanism of dispersion and from Smo125663 by the mechanism of wide genome duplication (WGD). One of the main characteristics of the Hevein domains is the presence of the eight highly conserved cysteine residues. At least one domain in the Hololectins contains all eight residues (Table 1, Figure 1).

### 3.2. Expression Profile of Hevein-like Genes in Different Organs

The transcription profile of Hevein-like members of the *S. moellendorffii* varies among members within and between the same tissue or organs analyzed. For instance, the expression level of Smo125663 in seeds, leaves, and shoots is ~four folds higher than in rhizophore. However, Smo99416, Smo99732, and Smo139127 expression levels are generally lower in all tissues or organs compared to the rest of the other Hevein-like genes. Moreover, the Smo99416 transcript is the only one that has a targeting cleavage site for miRNA silencing, and it is targeted by a single miRNA from the family MIR1082 (smo-niR1082a, located in scaffold 19) (see Supplementary File Figure S1 for more details). 

### 3.3. Structural Model Building of the Lectins and Their Secondary Structures

**Figure 2:**
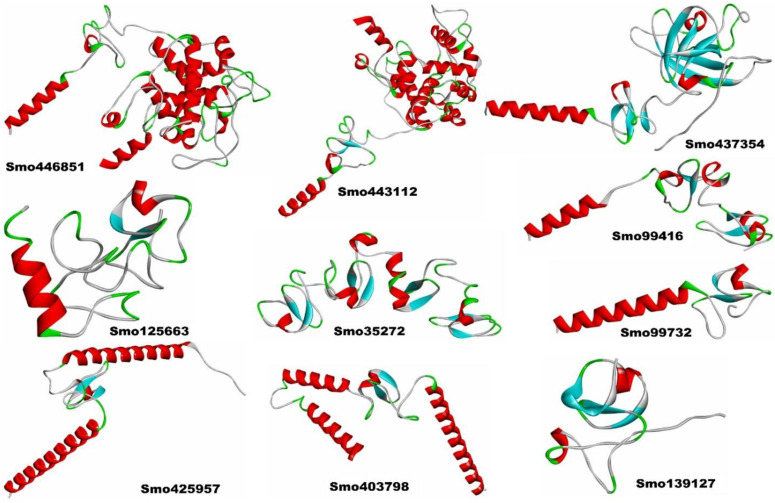
The 3D structural models of the Hevein-like lectins from *Selaginella moellendorffii*.

The 3D structural models of Smo446851 and Smo443112 lectin sequences were built with the restraints from both deep learning and homologous templates of known X-ray structures of the class-1 chitinase (Glyco_hydro_19) lectin; 2DKV [58], 3W3E [59], 4TX7 [60], 6LNR [61], and 1DXJ [62] using trRosetta webserver. Moreover, trRosetta proposed other templates for Smo437354 with confidence scores > 99, but rather low identity scores in relation to the templates Expansin-like proteins (PDB ID: 3D30) [63], Beta-expansin 1a (EXPB1) (PDB ID: 2HCZ_X) [64], Pollen allergen Phl p 1 (PDB ID: 1N10) [65], and cellulose binding proteins (PDB ID: 4JS7 and 4JJO) [66]. All templates were detected by running HHsearch against the PDB70 database, and confidence, coverage, identity, E-value, and Z-score scores were reported (Figure 2, also see Supplementary File Table S1). The other seven lectin model structures were constructed based on de novo folding guided by deep learning restraints using the trRosetta webserver. The TM scores for Smo446851 and Smo443112 were 0.88 and 0.86, respectively, whereas slightly lower scores ranging from 0.86 to 0.44 were predicted for the other lectins. The TM-score is a measure of confidence estimation that falls between 0 and 1, where high scores indicate a correctly predicted topology [38] (this is detailed in Supplementary File Table S2). The Ramachandran plots showed that >80% of the amino acid residues of Smo446851, Smo35272, Smo425957, Smo403798, Smo443112, Smo99416, Smo437354, and Smo99732 fell within the allowed region, whereas only 73.6% and 77.3% of the amino acids from the Smo125663 and Smo139127 were located within the allowed region. Furthermore, a very small percentage of the amino acids were located within the disallowed region, i.e., Smo446851 (0.8%), Smo125663 (2.8%), Smo403798 (0.9%), Smo443112 (0.8%), and Smo437354 (0.6%). However, these residues were not located within the Hevein domain and thus were not involved in the carbohydrate interaction (Supplementary File Figures S2 and S3A,B).

The analysis of the Hevein-like homolog sequences using SOPMA demonstrated the presence of α-helices (Hh), β-turns (Tt), random coils (Cc), and extended strands (Ec) (Supplementary File Figure S4). Almost all the lectins’ secondary structures exhibited a maximum frequency of random coils (Cc), except for Smo425957 and Smo403798, indicating high protein stability and flexibility [67]. Moreover, Smo425957 and Smo403798, with a higher proportion of α-helices, accounted for 54.24% and 39.52%, respectively. Alpha helices were more likely to be found in most thermophilic proteins [68].

### 3.4. Identifying the Binding Sites of the S Glycoprotein and Lectins for Macromolecular and Ligand Docking

In the receptor binding domain (RBD) of the S glycoprotein (PDB ID: 6VXX), the residues responsible for binding the ACE-2 are Thr470-Thr478, Tyr449, Leu455, Phe456, Phe486, Asn487, Tyr489, Gln498, Thr500, Asn501, and Tyr505 [69,70]. However, the amino acid residue Lys436, Gly465, Tyr468, Tyr472, Leu474, Phe475, Ala494, Phe505, Asn506, Tyr508, Gln512, Gly515, Gln517, Thr519, Asn520, Gly521, Tyr524, and the cofactor *N*-glycan NAG1321 (located at N165) were identified as the binding site for macromolecular (protein–protein) docking with lectins and ligand docking of cofactor NAG with the lectins, respectively. Details of the binding site residues of all the lectins considered for macromolecular docking were summarized in Supplementary File Table S3.

### 3.5. Molecular Docking of Lectins with SARS-CoV-2 Spike Protein

Since it has been previously established that spike protein monomer has 22 *N*-glycosylation 2 *O*-linked glycan points [15,71,72] and that Hevein-like lectins are specific for *N*-acetylglucosamine and chitin (polymer of GlcNAc) [73], 11 points of hybrid and complex *N*-glycans were of interest for the docking in this study. Due to their proximity to the ACE-2 RBD, the residues Asn165, Asn331, and Asn343 connected to complex *N*-glycan were of particular interest. The ACE-2 binding pocket and its surface *N*-glycans were studied for protein–protein and carbohydrate–protein interactions against each lectin, respectively.

We report that only three Hevein-like lectins (Smo35272, Smo425957, and Smo403798) interacted directly with the RBD residues in a protein–protein manner with binding free energy equal to −50.07, −16.12, and −22.72 Kcal/mol, respectively. However, neither of them interacted with the key residues of the receptor binding motif of the ACE2 (i.e., active key residues). Both Smo35272 and Smo403798 formed salt bridges with spike protein outside the vicinity of the RBD ((Smo35272; Arg9–Glu196 (3.2 Å), Arg47–Glu132 (3.3 Å), and Arg154–Glu298 (3.5 Å)) and (Smo403798; Arg72–Asp198 (3.2 Å))). Smo35272 formed the highest number of hydrogen bonds (20 bonds) with the spike protein; 40% of them were established within the proximity of the RBD (residue span 320–385). 

**Figure 3:**
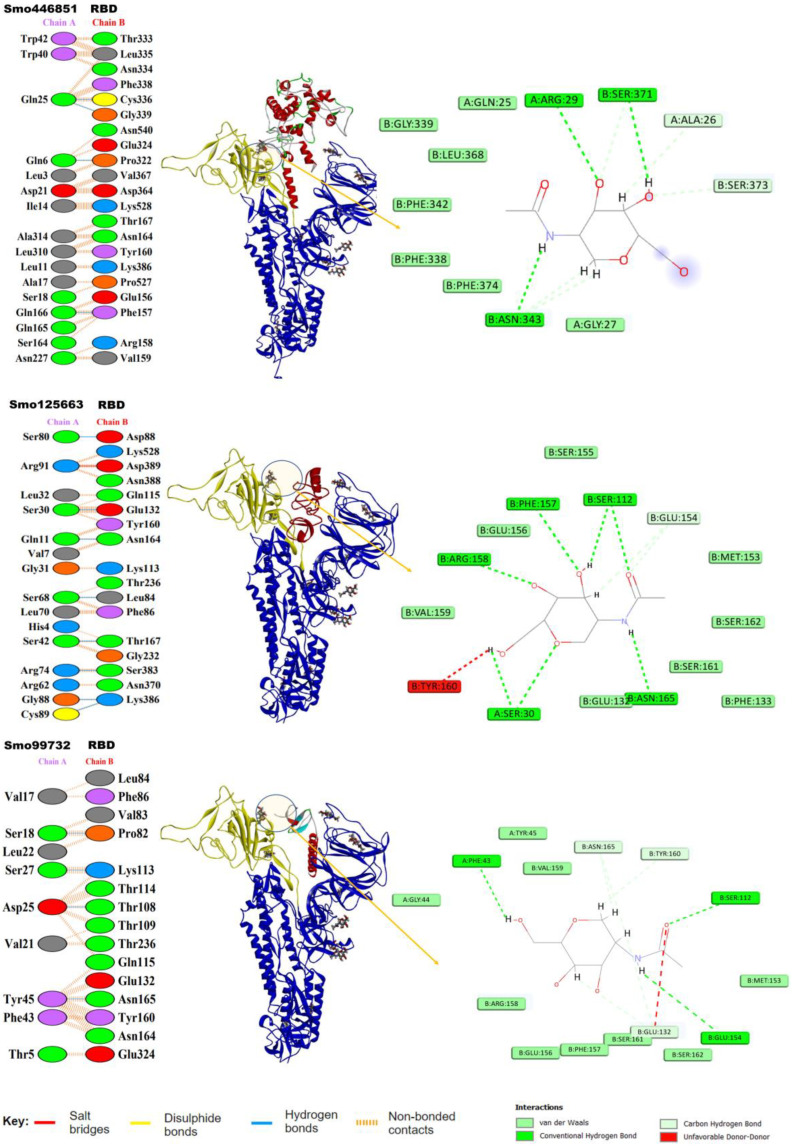
Lectins–S protein complexes and the interaction analysis using PBDSum. Lectin chains in red are designated as (Chain A), and the S protein in blue is designated (as Chain B); the RBD range 319–541 is highlighted in yellow. To the right is the network of hydrogen bonds (dashed lines) anchoring NAG to the amino acid residues in both chains A and B.

Our in silico molecular docking showed that only three lectins (Smo446851, Smo125663, and Smo99732) were able to interact with the NAG cofactors of the spike protein S1 via hydrogen bonding. In this, two hydrogen bonds were formed between Smo446851 and NAG1307 (located at Asn343), whereas a single hydrogen bond was observed between lectins Smo125663 and Smo99732, and the *N*-glycan NAG1321 (located at Asn165), respectively (Table 2). These Hevein-like lectins, which interact with the spike protein in a carbohydrate–protein manner, have also several other amino acids that interact directly with the residues located in the vicinity of the RBD. Among the 16 interacting residues of Smo446851, only Gln25 of the Hevein domain formed two hydrogen bonds with the residues from the spike protein: Cys336 and Gly339. Based on the PPI generated by the PDBSum server, 12 of the 15 interacting residues from Smo125663 located in the Hevein-domains (region 19–49 and 57–91) showed good interaction with the RBD residues, of which three hydrogen bonds were formed with two residues from RBD (Ser383 and Lys386). Moreover, a salt bridge interaction was also reported between Arg91 from Smo125663 and ASP389 from the RBD. A lower number of interacting residues were observed for Smo99732 (nine amino acids), and only two residues of which Phe43 and Tyr45 are in the Hevein domain. Tyr45 shared a hydrogen bond with key residue Asn165 from the RBD (a glycosylation point for complex *N*-glycan) (Figure 3). Furthermore, the estimated Generalized Born Model and solvent accessibility (MM/GBSA) calculations of lectins with S glycoprotein of docked complexes provided predictions for the binding energy and detailed the contributions to binding free energy per residue to assist in the analysis of binding structures in proteins by considering the electrostatic potentials (ELE), the Van der Waals potentials (VDW), the polar solvation (GB), and the nonpolar contribution to the solvation (SA) [48]. The Smo99732, which is complexed with S protein, has a lower MM/GBSA score (−26.5 Kcal/mol) compared to the complexes with Smo446851 (−17.5 Kcal/mol) and Smo125663 (−13.0 Kcal/mol). No favorable interactions were detected between the interactive key residues at the RBM or the RBD and the rest of the tested Hevein-like lectins (i.e., Smo443112, Smo99416, Smo437354, and Smo139127); hence, they were excluded from further analysis.

It is evident that only three lectins (Smo446851, Smo125663, and Smo99732) demonstrated a superior ability to interact with spike RBD. Consequently, we attempted to investigate their potential antiviral activity by performing a simultaneous docking of multi-body complexes of receptor-binding domain (RBD) of the SARS-CoV-2 spike protein, lectins (Smo446851, Smo125663, and Smo99732), and ACE2 which has been identified as the major cell entry receptor for SARS-CoV2 [74].

**Figure 4:**
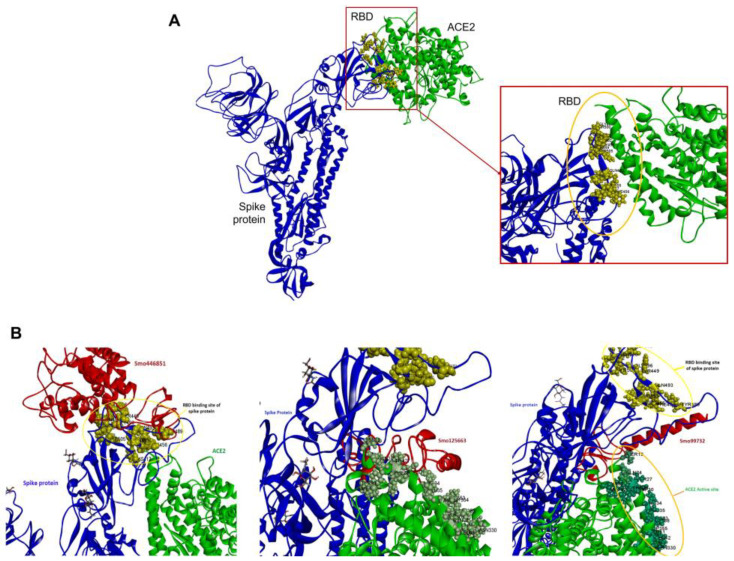
Docking of ACE to free and lectins–RBD complexes. (**A**) ACE2–RBD complex. (**B**) ACE2–Lectins–RBD complexes. The SARS-CoV-2 (Chain B, in blue), lectins Smo446851, Smo125663, and Smo99732 (Chain C, in red), and ACE2 (Chain C, in green). The residues in the RBD (yellow sphere) that are involved in the interaction include Lys417, Gln493, and Asn501, while the residues in the NTD of ACE2 include ASP30, His34, Lys353, and Tyr354.

Initially, ACE2 was docked onto the RBD of spike protein and has shown favorable interactions with the residues of RBD Tyr505, Gly502, Gly496, Asn501, Thr500, Gln498, Tyr449, Gly446, Phe456, Lys417, Gln493, Leu455, and Tyr453 with free binding energy MM/GBSA −18.57 Kcal/mol. Afterward, a multi-body docking run conducted using the HADDOCK server revealed that the lectins are embedded between the RBD and the active site *N*-terminal domain (NTD) of ACE2. This leads to interference with RBD–ACE2 interactions (Figure 4A). Lectins (Smo446851 and Smo99732) interact with the receptor-binding domain (RBD) of the spike protein through a combination of hydrogen bonds and van der Waals interactions with HADDOCK binding energy equal to −387.01 and −282.609 Kcal/mol, respectively. In comparison, lectin Smo125663 (binding energy −716.255 Kcal/mol) showed favorable interactions with the residues of the N-terminal domain (NTD) of ACE2 (Figure 4B).

### 3.6. Hotspot Analysis of RBD–Lectin Complex

Using Knowledge-based FADE and Contacts 2 (KFC-2), the hotspot residues and/or clusters were predicted at the interface between S protein and lectins. The values from the KFC-a model were used as their predictability outcomes that were superior to those of the KFC-b model because it is based on interface solvation, atomic density, and plasticity features, as indicated earlier [53]. Specifically, residues Gln25, Asp21 from Smo446851, Smo125663 residues Arg91 and Gln11, and the Smo99732 Asp25 were identified as hotspot residues and reported higher KFC-2-a scores compared to other residues (Supplementary File Figure S5). Complimentary hotspot residues Val367, Asp364, Lys386, Gln115, and Thr109 were identified as hotspot residues on S glycoprotein.

### 3.7. Normal-State Analyses via Torsional Coordinate-Association

**Figure 5:**
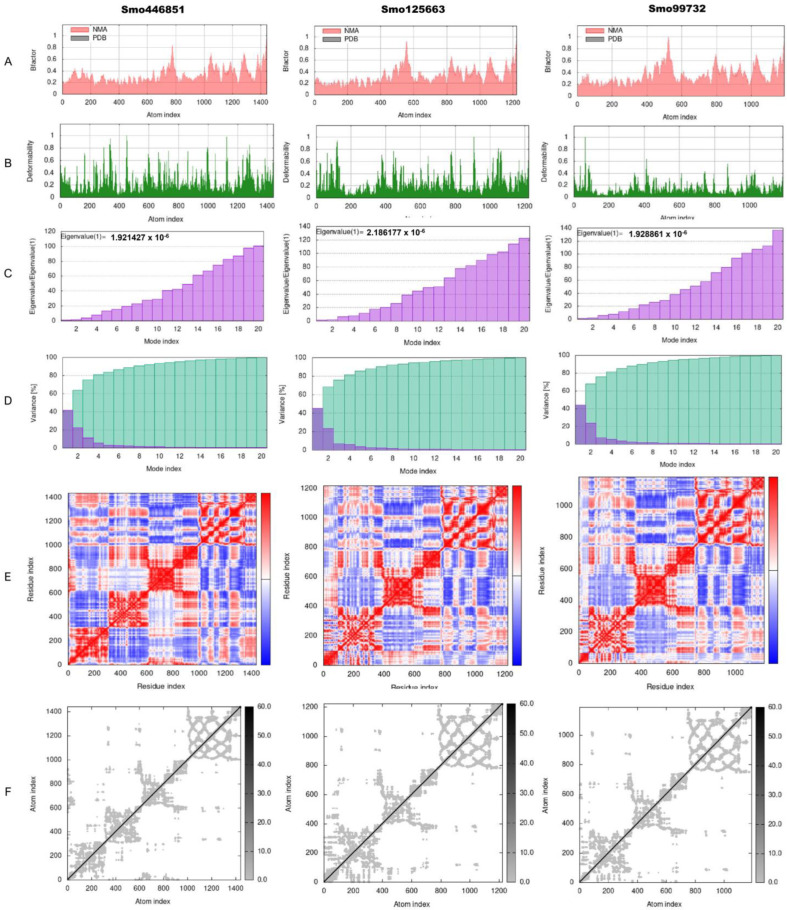
Normal-state analyses via iMODS server for the docked lectins (Smo446851, Smo125663, and Smo99732)–spike protein complexes. (**A**) B-factor indices, (**B**) deformability plot (the peaks indicated the non-rigid regions of the complexes), (**C**) eigenvalues, (**D**) variance plot (individual variances are purple, while cumulative variances are green), (**E**) covariance map (correlated (red), uncorrelated (white), or anti-correlated (blue) motions), and (**F**) elastic network analyses (darker grey regions indicate stiffer regions of the complex).

The torsion angle-related normal state analysis of lectins (Smo446851, Smo125663, and Smo99732) docked onto the spike glycoprotein was performed using the iMODS server to examine their inherited stability and conformational mobility (Figure 5). The B-factor is correlated to the atom’s displacement around the conformational equilibrium. The three lectin complexes, each with S-protein, have the highest flexibility and large atomic displacements around the atomic positional range (400–600) (Figure 5A). The complex deformability index, which indicates higher individual distortions for the docked complexes, showed general steady binding characterized by minimum deformities at coordination within the range (0–1 Å), and the peaks represent the location of hinges; the Smo99732–S protein complex is more rigid than the other two complexes (Figure 5B). Considering that the energy required to distort the complex is proportional to its eigenvalue, the lower the eigenvalue, the easier the complex is to deform [75]. The estimated eigenvalues representing the motion stiffness for each lectin–spike protein complex were 1.92 × 10^−6^, 2.12 × 10^−6^, and 1.93 × 10^−6^ for Smo446851, Smo125663, and Smo99732 complexes, respectively (Figure 5C). The eigenvalues were inversely proportional to variance, predicting the significantly higher mobility of the lectins compared to the spike protein across collective functional motion (Figure 5D). The iMODS server provided a covariance matrix depicting uncorrelated (white), correlated (red), and anti-correlated (blue) residue pairs. The three docked complexes have high correlated residue-pair motion compared to the uncorrelated residue-pair motion (Figure 5E). The elastic-network model describes the lectin–spike protein complex’s distinct flexibility patterns. It visualizes the atom pairs connected via springs by illustrating them according to their stiffness degrees. Dark grey is usually associated with stiffer strings. The dots indicate one spring, and a grey area indicates stiffer springs (Figure 5F). Overall, the docked complexes showed stable binding, minimum deformities, and complex rigidity.

### 3.8. Molecular Dynamics Simulation

**Figure 6:**
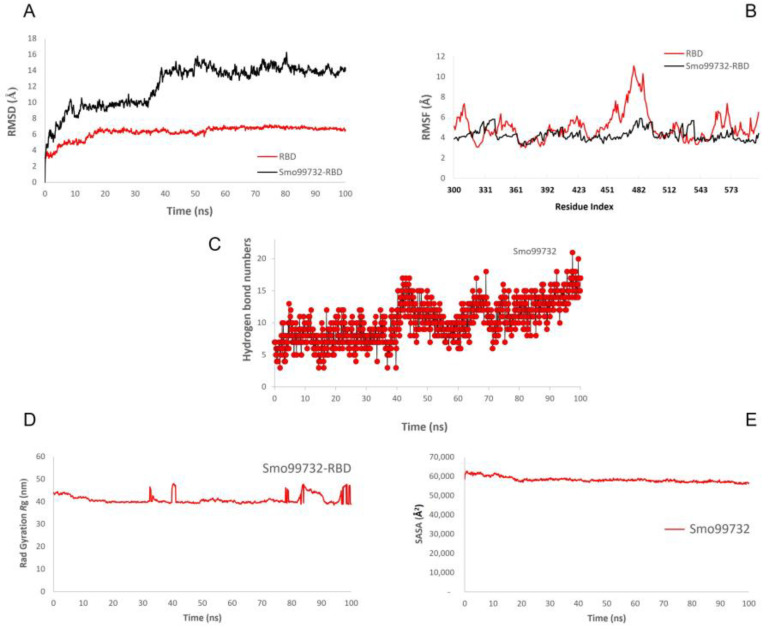
Molecular dynamic simulation (MDS) for the spike protein’s RBD complexed with Smo99732. (**A**) RMSD, (**B**) RMSF, (**C**) number of hydrogen bonds, (**D**) radius gyration, and (**E**) SASA.

The molecular dynamics simulations were performed to evaluate the stability of RBD of SARS-CoV-2 with lectins (Smo446851, Smo125663, and Smo99732) at 100 ns. The simulations were carried out for spike protein’s RBD in its free form and in complex with Smo446851, Smo125663, and Smo99732 to evaluate the protein’s motions and flexibility, which contribute to the interaction dynamics of complexes. The Desmond simulation trajectories were analyzed, and the root mean square deviation (RMSD), root mean square fluctuation (RMSF), and RBD–lectins contacts were calculated (Figure 6, Supplementary File Figure S6). During the initial period of RBD–lectins (Smo446851 and Smo99732) MD simulation, the deviation in the complex was not drastic and remained below 10 Å and 9 Å, respectively. While RBD-Smo125663-lectin-3 showed relative fluctuations as the RMSD declined from 11 Å to 7 Å at 21 and 36 ns, then equilibrated at 16 Å. Meanwhile, the RMSD of Cα atoms of the backbone of the RBD underwent fewer fluctuations than RBD–lectins complexes throughout the simulation. The Root Mean Square Fluctuation (RMSF) was utilized to further investigate the stability of the complexes formed by docked RBD–lectins (Smo446851, Smo125663, and Smo99732). RMSF was measured to compute the residual flexibility over 100 ns. The RBD–lectins complexes are less flexible compared to the free RBD. The radius of gyration (*R*g) was used to study the ligand’s compactness and stability. Throughout the simulations, the *R*g plots for RBD–lectins complexes indicated relatively fewer fluctuations except for the RBD-Smo446851 complex; its *R*g trajectory reached equilibration at ~ 75 ns and was steady between 75 and 100 ns, indicating that the ligand is effectively fitted at the active site of the spike protein. RBD–lectins (Smo125663 and Smo99732) were relatively stable over the time of the simulations. However, both complexes displayed relatively lower *R*g values than the RBD-Smo446851 complex, making a structure rigid with a restricted rotation figure. SASA plots for RBD–lectins complexes showed stable fluctuations over the time of the simulations (Figure 6). During the simulation run time, Smo99732 formed 4–17 hydrogen bonds with the active site residues of RBD, Smo446851 (6–20), and Smo125663 (5–20), and the later established more hydrogen bonds compared to the other complexes.

### 3.9. Mutant Spike Protein Interaction with Lectins

**Figure 7:**
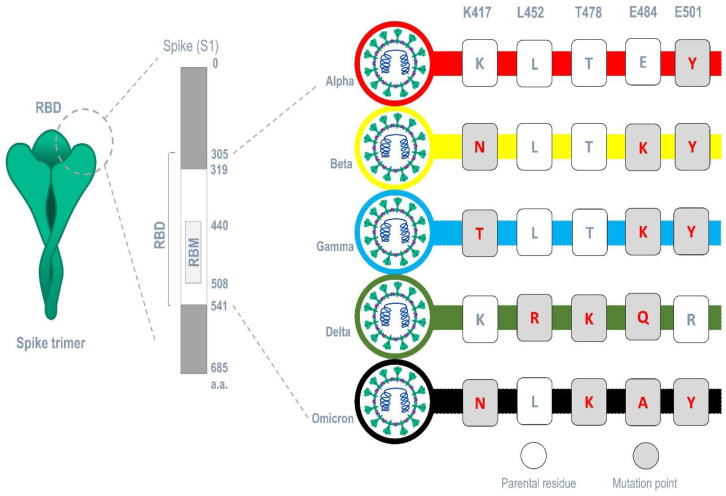
Common mutations occurring in major SARS-CoV-2 variants within the region of the RBM.

The carbohydrate-interacting lectins Smo446851, Smo125663, and Smo99732 were studied for interaction with different spike protein mutants commonly occur in SARS-CoV-2 variants (alpha, beta, gamma, delta, and omicron) (Figure 7). Five mutations related to the RBM region were selected, and five mutant 6VXX structures were produced, one for each mutation. Analyzing the 15 generated docked complexes revealed that almost all the RBM regions were now accessible for the bind of Hevein-like lectins. Additionally, many forms of interactions, including the formation of hydrogen bonds, were evident. Ile468, which is one of the residues required for the binding of the ACE-2 receptor, is mainly targeted by lectins in 11 complexes related to all mutations (Supplementary File Table S4). However, Smo125663 and Smo99732 did not interact with N-glycan complexes, and the interaction was solely in a protein–protein manner. Smo446851 retained its carbohydrate–protein interaction with NAG1307 in mutant Leu452Arg, and NAG1306 in mutants Thr478Lys and Asn501Lys (Supplementary File Figure S7). The calculated MM/GBSA free binding energy for Smo446851-mutant Glu484Lys complex was higher than reported for parent RBD and other mutant complexes (−29.16 Kcal/mol). Smo125663 showed relatively poor interaction among the mutant complexes, and was compared to its interaction with parent RBD, while Smo99732 maintained the highest free binding energy score in all mutant complexes except for the Glu484Lys-RBD complex (−8.93 Kcal/mol). 

Furthermore, the analysis of spike proteins that carry different sets of mutation points in their RBM that correspond to each variant (alpha (E501Y), beta (K417N, E484K, and E501Y) gamma (K417T, E484K, and E501Y), delta (L452R, T478K, and E484Q), and omicron (K417N, T478K, E484A, and E501Y)) (Figure 7) revealed that Smo125663 have better free binding energy with both variants alpha (−31.8 Kcal/mol) and beta (−21.49 Kcal/mol) compared to the other lectins. It only interacts in a protein–protein manner with the RBD by forming six hydrogen bonds. The three lectins bind the RBD of the variant omicron in relatively low free binding energies. The carbohydrate–protein binding was observed for the interaction between Smo46851 active residue Thr49 of the lectin domain and the alpha mutant S protein’s NAG1306 at 2.94 Å instead of the NAG1307 interaction reported for the parent spike protein (Table 3).

## 4. Discussion

### 4.1. Selaginella Moellendorffii Hevein Lectins

The spike moss *Selaginella moellendorffii* is a member of the lycophytes, nowadays classified under one of the three surviving families (Selaginellaceae). Its genome is the smallest reported plant genome, with a size of ~100 Mbp [21]. The *S. moellendorffii* is a genetic model being utilized as a key to understanding how vascular plants evolved and gained many beneficial traits. Evolutionary studies showed that the *S. moellendorffii* genome harbors gene members belonging to all documented plant lectin families except for the *Agaricus bisporus* agglutinin (ABA) family [76]. The functional plasticity of plant lectins allows them to participate in various endogenous biological processes as well as defense molecules against predators and pathogens, hence their wide application in medicine and pharmaceutical fields. With the emergence of high throughput genome sequencing, the availability of genomic source databases, datasets, and their translation, and tools, novel sources of bioactive peptides and proteins were easy to identify and study. Hevein-like lectins are defense molecules; their activity is mediated by the interaction with pathogenic microorganisms’ surface chitin or its derivatives *N*-acetylglucosamine [77]. Since the start of the COVID-19 pandemic, scientists have been searching for new drugs and vaccines to combat the virus. Many plant lectins inhibit SARS-CoV-2 by targeting the spike protein either through the interaction with the complex or high-mannose *N*-glycans found in the vicinity of the RBD or by interacting with the RBD residues through direct protein–protein binding [17,18]. Lectins from the Hevein (chitin-binding lectins) family mined from the *S. moellendorffii* genome were studied for both binding modes to the RBD of the spike protein. 

### 4.2. Hevein Lectin Interaction with SARS-CoV-2 Spike Protein’s RBD

Three Hevein-like lectins (i.e., Smo35272, Smo425957, and Smo403798) bind in the RBD region of the spike protein that spans from residues 319 to 541 in a protein–protein manner. However, none of these lectins formed any interaction with the residues that constitute the ACE2 receptor binding motif (residues 438–508, Tyr505, Gly502, Gly496, Asn501, Thr500, Gln498, Tyr449, Gly446, Tyr489, Phe456, Lys417, Gln493, Leu455, and Tyr453). These lectins bound deeply within the groove located at the top of the spike protein while some residues interacted with the amino acids flanking the RBM, especially in the region 300–399. Unlike the interaction reported for the Urtica dioica Agglutinin (UDA), examined for binding RBD (6VXX), UDA bound 14 residues (i.e., Tyr505, Gly502, Gly496, Asn501, Thr500, Gln498, Tyr449, Gly446, Tyr489, Phe456, Lys417, Gln493, Leu455, and Tyr453) which are also involved in the RBD–ACE2 interaction [19]. Therefore, the binding of Smo35272, Smo425957, and Smo403798 needs further investigation to understand how these lectins can influence and interfere with the binding of ACE2. 

Porto et al. (2012) studied the binding of Smo99732 (GenBank ID: XP 002973523) devoid of the signal peptide sequence (23 amino acids) with *N, N, N*- triacetyleglucosamine (GlcNAc)3. Three of these amino acids, Phe20, Tyr22, and Tyr29, were responsible for the binding stabilized with hydrogen bonds during most of the simulation time [78]. These residues are conserved as part of the Hevein domain active site. However, the interaction with the RBD NAG1321 and NAG1307 via hydrogen bonds involved a different set of amino acids (Smo99732–Phe43, Smo125663–Ser30, Smo446851–Ala26, and Arg29). The Asn343, along with Asn282 and Asn331, act as a shield that covers and protects the spike protein’s RBD against neutralizing antibodies. They are also involved in the binding of the ACE2-RBD. Mutations or blocking of these glycosides are reported to influence viral binding and significantly reduce its infectivity [9]. Lokhande et al. (2022) investigated the binding of several lectins to the RBS of the S protein. Only NPA *Narcissus pseudonarcissus* Agglutinin and UDA, a Hevein-like lectin, interacted with GlcNAc complex *N*-glycans. However, UDA, a Hololectin with two tandemly arrayed Hevein domains, shared 34.62% homology with Smo125663. Both lectins interact with the *N*-glycan through the formation of hydrogen bonds between the first domain and the complex at 2.24 and 2.5 Å, respectively. However, UDA interacts with NAG1322, while Smo125663 interacts with NAG1321, and both complexes also interact with RBD Asn370 [71].

### 4.3. Interaction with Mutant SARS-CoV-2 Spike Protein’s RBD

Beta, gamma, delta, and omicron are classified as variants of concern (VOC) due to their increased transmissibility, disease severity, immune response impact, and drug efficacy [79]. Several mutations occur in the RBM of the spike protein’s RBD region, endowing the virus with enhanced ACE2 receptor binding and/or better antibody evasion [80,81]. By neutralizing the free virions, *Triticum vulgaris* agglutinin (WGA) has been shown to prevent infection of SARS-CoV-2 and its variants alpha and beta [82]. In an in vitro experiment employing Vero E6 cell lines, Griffithsin (GRFT) was found to bind to the spike protein and thereby prevent SARS-CoV-2 and its variants delta and omicron from entering the cells [83]. The NTL-125, another lectin, was investigated for its high affinity binding to the RBD of several variations. Such binding occurs through the α-helical tail of the protein, which interacts with glycan moieties and increases the binding strength [84]. Considering our findings, Smo99732 appears to be a promising candidate for carbohydrate–protein interactions with wide-type spike glycoproteins, given its complex stability, low free energy compared to other *S. moellendorffii* Hevein-like lectins, and capacity for improved protein–protein interactions with mutant variants’ RBMs.

## 5. Conclusions

This comprehensive in silico investigation provided a novel contribution among other published studies and confirmed the effectiveness of plant mannose- and GlcNAc-specific lectins against RNA viruses, including HIV, SARS-CoV, and SARS-CoV-2. The latter two are inhibited by lectin-viral spike *N*-linked glycoprotein interactions. In the current study, by genome-wide search, we report on ten novel chitin-specific Hevein-like lectins from Selaginella moellendorffii, three of which, Smo446851, Smo125663, and Smo99732, were able to interact favorably with the receptor binding domain (RBD) of the spike protein. The binding specificity of these lectin homologs with spike-RBD can be further investigated using in vivo systems, such as the Vero cell line. Additionally, given the current SARS-CoV-2 mutation frequency, we anticipate that *S. moellendorffii* lectin-based medications, specially Smo99732, might be a candidate against newly emerging variants, such as alpha, beta, gamma, and omicron that are responsible for the rapid spread of COVID-19. Lectins are easier and highly feasible candidates than molecular vaccines, albeit toxicological analysis to address potential reactions is imperative.

## Figures and Tables

**Table 1:** Characterization of Hevein-like homologs from *Selaginella moellendorffii*.

ID	Scaffold #	*pI*	MWt (KDa)	SP	TM	Targeting Class	*N*-Glycan	*O*-Glycan
Smo446851	79	6.43	34.364	Sec/SPI	0	SP	−ve	+ve
Smo35272	30	8.62	15.603	Other	0	IC	−ve	+ve
Smo125663	79	8.47	10.046	Other	0	IC	−ve	+ve
Smo425957	79	8.02	12.554	Sec/SPI	1	SP	−ve	−ve
Smo403798	1	8.6	13.395	Sec/SPI	1	SP	+ve	−ve
Smo443112	30	6.79	34.635	Sec/SPI	0	SP	−ve	+ve
Smo99416	21	5.79	10.038	Sec/SPI	0	SP	−ve	−ve
Smo437354	0	5.75	20.944	Sec/SPI	0	SP	+ve	+ve
Smo99732	22	8.09	6.57	Sec/SPI	0	SP	−ve	−ve
Smo139127	757	8.07	6.204	Other	0	IC	−ve	−ve

*pI*: isoelectric point, SP: signal peptide, TM: transmembrane domain, IC: intracellular., −ve: negative, +ve: positive.

**Table 2:** The interacting residues of spike protein (PDB: 6VXX_B) at RBD bound with lectins.

Type of Interactions	Interacting Residues of S Protein	Lectins	Interacting Residues of Lectins	Distance (Å)	Docking Score ●	Confidence Score *	MM/GBSA ●
H-bond	NAG 1307	Smo446851	Ala26	2.8	−182.44	0.66	−17.49
	NAG 1307		Arg29	2.7			
H-bond	Lys386	Smo35272	Gln110	2.2	−163.59	0.568	−50.07
	Thr385		Gln94	2.0			
	Asn370		Gln80	1.9			
	Ser366		Gln80	2.2			
	Val320		Arg122	2.4			
H-bond	NAG 1321	Smo125663	Ser30	2.5	−160.85	0.55	−13.03
H-bond	Asn370	Smo425957	Ser61	1.9	−197.02	0.72	−16.12
	Lys386		Cys38	2.5			
	Lys386		Pro36	2.4			
	Ser383		Tyr58	3.1			
	Ser325		Arg46	2.5			
H-bond	Tyr369	Smo403798	Arg49	2.2	−187.76	0.68	−22.72
H-bond	NAG 1321	Smo99732	Phe43	2.6	−136.67	0.53	−26.45

● Docking score and the free energy MM/GBSA are calculated in Kcal/mol. * Confidence score > 0.7, the two molecules would be very likely to bind; between 0.5 and 0.7, the two molecules would be possible to bind; and <0.5, the two molecules would be unlikely to bind.

**Table 3:** The hydrogen bonds interaction formed between residues from the variants RBD of the S protein RBD and lectins.

Mutant	Lectin	# of H-Bonds	Interacting Residues		Docking Score ●	Confidence Score *	MM/GBSA ●
			S Protein	Lectins			
Alpha	Smo446851	6	Tyr351, Ser359, Asn360, **NAG1306**	Ser6, Tyr38, Thr48, **Thr49, Ala51**	−212.92	0.7788	−23.64
	Smo125663	6	Glu340, Thr345, Ser349, Ser359, Arg457, Arg466	Tyr39, Gln22, Arg59, Asn6, Glu83, Ser44	−196.60	0.7175	−31.8
	Smo99732	4	Glu340, Arg357, Ser359	Trp167, Gln195, Ser194	−168.16	0.5895	−9.22
Beta	Smo446851	7	Asn331, Thr333, Thr581, **NAG1306**	Ser108, Ser321, Asp234, Ser61, **Pro33, Asn111**	−161.94	0.5594	−6.31
	Smo125663	6	Trp353, Arg355, Asn448, Arg466, Thr470	Asn67, Ser30, Gly50, Tyr84	−196.15	0.7157	−21.49
	Smo99732	7	Tyr351, Ser359, Asn360, **NAG1306**	Thr48, Ser6, Tyr38, Thr48, **Thr49, Ala51**	−168.65	0.5922	−8.67
Gamma	Smo446851	10	Tyr369, Thr415, Phe456, Arg457, Ser459, Gln474, Thr478, Asn481, Tyr505	Leu13, Ala225, Gln166, Gln195, Leu191, Gln165, Gln166, Asn202, Gln316	−220.86	0.8049	−35.31
	Smo125663	6	Trp353, Arg355, Arg454, Arg466, Thr470	Asn67, Ser42, GLy50, Tyr84	−197.62	0.7216	−15.04
	Smo99732	5	Phe347, Arg357, Asn450, Leu492	Gln24, Ser6, Tyr38, Ala23, Thr48	−218.65	0.7979	−25.68
Delta	Smo446851	4	Ser459, Asn481, Thr457, Gln755	Gln195, Asn202, Asp48, Ser296	−183.89	0.6632	−6.6
	Smo125663	5	Ala352, Asn450, Ser469, Thr470	Arg21, Gln22, Tyr93, Arg91	−202.50	0.7408	1.6
	Smo99732	5	Phe347, Ala352, Arg357, Asn450, Pro561	Gln24, Lys20, Ser6, Tyr38, Lys2	−208.37	0.7627	−28.0
Omicron	Smo446851	6	Tyr369, Lys378, Ser383, Ser459, Asn481, Gln755	Leu13, Ala19, Ala17, Gln195, Asn202, Ser296	−217.22	0.7932	−16.22
	Smo125663	5	Arg457, Arg466, Ile464, Glu516	Glu83, Ser44, Gln36, Tyr84, Asn6	−185.52	0.6705	−22.37
	Smo99732	7	Phe347, Arg357, Ser359, Asn450, Leu492	Gln24, Ser6, Tyr38, Ala23, Thr48	−219.82	0.8016	−25.94

● Docking score and the free binding energy MM/GBSA are in Kcal/mol. * Confidence score > 0.7, the two molecules would be very likely to bind; between 0.5 and 0.7, the two molecules would be possible to bind; and <0.5, the two molecules would be unlikely to bind. Bold entries represent the hydrogen bond formed between the carbohydrate NAG and its lectin counterpart residues.

## Data Availability

All data generated and analyzed during this study are included in the main article, and its supplementary data are provided in the supplementary section of this manuscript. Genomic data of *Selaginella moellendorffii* were publicly available and freely accessible, and all related UTR links were provided within the article under relevant mentions.

## Supplementary Materials

The following supporting information can be downloaded at https://www.mdpi.com/article/10.3390/cimb45070372/s1. Table S1: trRosetta templates used for lectin homology modeling; Table S2: Predicted TM-score for each lectin model; Table S3: The binding site residues of the lectins; Table S4: The hydrogen bonds formed in protein–protein interactions of mutant S protein RBD and lectins; Figure S1: Expression profile of Hevein-like genes from Selaginella moellendorffii in different tissues/organs; Figure S2: The Ramachandran plots for the Hevein-like lectin model structures; Figure S3A,B: The quality and Z-score of the Hevein-like lectin structural models (five models); Figure S4: Prediction of the secondary structure of Hevein-like lectins from Selaginella moellendorffii. Hh: α-helices, Tt: β-turns, Cc: random coil, and Ec: extended strand; Figure S5: Hotspot residue scores predicted by KFC-2 for the PPI involving SARS-CoV-2 S glycoprotein; Figure S6: Molecular dynamic simulation (MDS) for the spike protein’s RBD complexed with Smo446851 and Smo125663. (A) RMSD, (B) RMSF, (C) number of hydrogen bonds, (D) radius gyration, and (E) SASA; Figure S7: NAG1306 and NAG1307 of mutant spike proteins interactions with Smo446851 Hevein domain active residues.
