# Supplementary material for: Genome-Wide Mining of Selaginella moellendorffii for Hevein-like Lectins and Their Potential Molecular Mimicry with SARS-CoV-2 Spike Glycoprotein
Source: Curr Issues Mol Biol. 2023 Jul 14;45(7):5879–901. doi: 10.3390/cimb45070372 (PMC10378081; doi:10.3390/cimb45070372)
Supplement: Supplementary file 1 [file cimb-45-00372-s001.zip › cimb-2377180-supplementary.pdf]

Genome-wide mining for Hevein-like lectins from *Selaginella moellendorffii* and their potential molecular mimicry with SARS-CoV2 spike glycoprotein

Supplementary File S1

Tables

Table S1: trRosetta templates used for lectin homology modeling

ID	Template	Confidence	Coverage	Identity	E-value	Z-score
Smo446851	2DKV_A	100.0	89.9	62.9	2.9E-34	63.357
	6LNR_A	100.0	88.3	54.1	8.5E-36	65.895
	3W3E_A	99.9	75.6	64.0	1.3E-21	42.420
	4TX7_A	99.8	77.2	62.9	2.6E-20	40.244
	1DXJ_A	99.9	75.9	62.0	1.7E-22	43.870
Smo443112	2DKV_A	100.0	88.8	63.6	9.2E-35	63.931
	6LNR_A	100.0	88.4	54.5	2.2E-35	63.921
	3W3E_A	99.9	74.7	64.5	7.2E-22	42.712
	4TX7_A	99.8	76.2	62.9	1.5E-20	40.581
	1DXJ_A	99.9	75.0	62.4	1.6E-22	43.822
Smo437354	3D30_A	99.8	62.5	25.0	2.3E-19	33.403
	1N10_A	99.7	71.0	23.0	9.3E-19	32.541
	2HCZ_X	99.7	71.5	28.5	9.5E-19	32.514
	4JS7_A	99.7	60.5	18.0	1.4E-18	32.253
	4JJO_A	99.7	60.5	21.0	2.7E-18	31.887

Z-score: Normalized score of the raw alignment. Z-score > 11 indicates a reliable hit, E-value: values closer to 0 indicate a more significant hit.

Table S2: Predicted TM-score for each lectin model.

ID	TM-score	ID	TM-score
Smo446851	0.879	Smo443112	0.858
Smo35272	0.467	Smo7_99416	0.590
Smo125663	0.520	Smo437354	0.740
Smo425957	0.443	Smo99732	0.722
Smo403798	0.513	Smo139127	0.689

Table S3: The binding site residues of the lectins

Lectin	Hevein domain	Active residues in the binding site
Smo446851	22-58	S38, W40, G41, W42, G44, D48, H49
Smo35272	1-38, 39-76, 77-113, 120-154	S93, F95, G96, Y97, G99, F104, S136, F138, G139, Y140, G142, Y147
Smo125663	19-49, 57-91	S73, F75, G76, Y77, G79, Y84
Smo425957	38-68	S54, F56, G57, Y58, G60, Y65
Smo403798	38-72	S54, F56, G57, Y58, G60, A64, F65
Smo443112	22-58	S38, W40, G41, W42, G44, D48, H49
Smo99416	23-59, 44-99	S39, Y41, G42, Y43, G45, Y50
Smo437354	26-57	S37, W39, G40, Y41, G43, Y48
Smo99732	30-62	S41, F43, G44, Y45, G47, Y52
Smo139127	19-53	S35, F37, G38, Y39, G41, Y46

Table S4: The hydrogen bonds formed in protein-protein interactions of mutant S protein RBD and lectins.

**Mutant**	**Lectin**	**# of H-bonds**	**Interacting residues**		**Docking score●**	**Confidence score***	**MM/GBSA ●**
			**S protein**	**Lectins**			
K417N	Smo446851	5	Leu335, Lys444, Asn450, Arg466	Gln316, Ser108, Glu112, Gln166	-182.71	0.66	-11.11
	Smo125663	8	Thr345, Ser349, Tyr351, Arg355, Tyr449, Leu452, Arg466	Arg74, Glu,83, Thr81, Gln36, Ser82, Glu83, Ser44	-179.18	0.64	-11.70
	Smo99732	5	Ser349, Asn354, Asp467, Ser469	Tyr45, Asp25, Tyr52, Thr48	-193.45	0.70	-32.78
L452R	Smo446851	4	Thr333, Thr345	Gly244, Glu112, Lys239	-177.70	0.64	-14.48
	Smo125663	2	Thr345, Phe347	Gln22, Arg59	-177.76	0.64	-06.97
	Smo99732	2	Arg453, Glu516	Gln61, Thr5	-186.23	0.67	-31.41
T478K	Smo446851	1	Leu582	Pro33	-197.06	0.72	-9.54
	Smo125663	7	Thr345, Trp353, Arg355, Arg454, Arg466, Thr470	Arg27, Asn67, Ser42, Gly50, Tyr84	-206.57	0.76	-19.55
	Smo99732	5	Ser349, Asp467, Ile468, Ser469	Tyr45, Tyr52, Thr48	-195.79	0.71	-34.52
E484K	Smo446851	6	Asn540, Asn542, Asp745, Glu748, Asp843	Arg4, Gln165	-201.87	0.74	-29.16
	Smo125663	5	Thr354, Arg454, Arg466, Thr470	Arg27, Ser42, Gly50, Tyr84	-201.53	0.74	-16.47
	Smo99732	1	Tyr449	Lys2	-164.55	0.57	-8.93
E501Y	Smo446851	0	—	—	-166.31	0.58	9.94
	Smo125663	2	Ser359, Asn450	Glu9, Arg74	-207.24	0.76	-10.63
	Smo99732	3	Asn354, Arg357, Asn360, Lys444	Lys20, Ser6	-205.90	0.75	-28.79

●Docking score and the free binding energy MM/GBSA are in Kcal/mol

*Confidence score > 0.7, the two molecules would be very likely to bind; between 0.5 and 0.7, the two molecules would be possible to bind; < 0.5, the two molecules would be unlikely to bind.

Figures

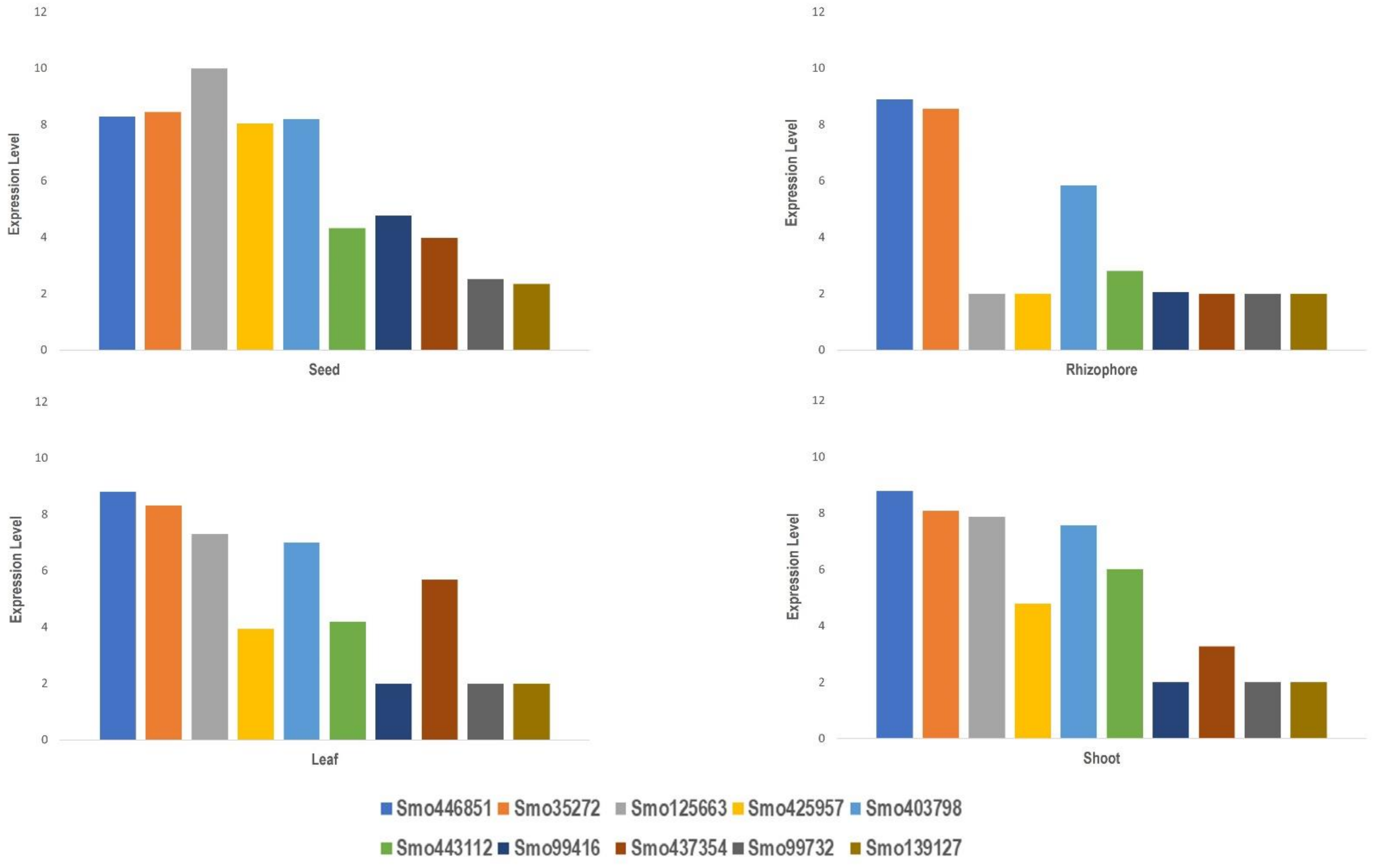

Figure S1: Expression profile of Hevein-like genes from Selaginella moellendorffii in different tissues/organs.

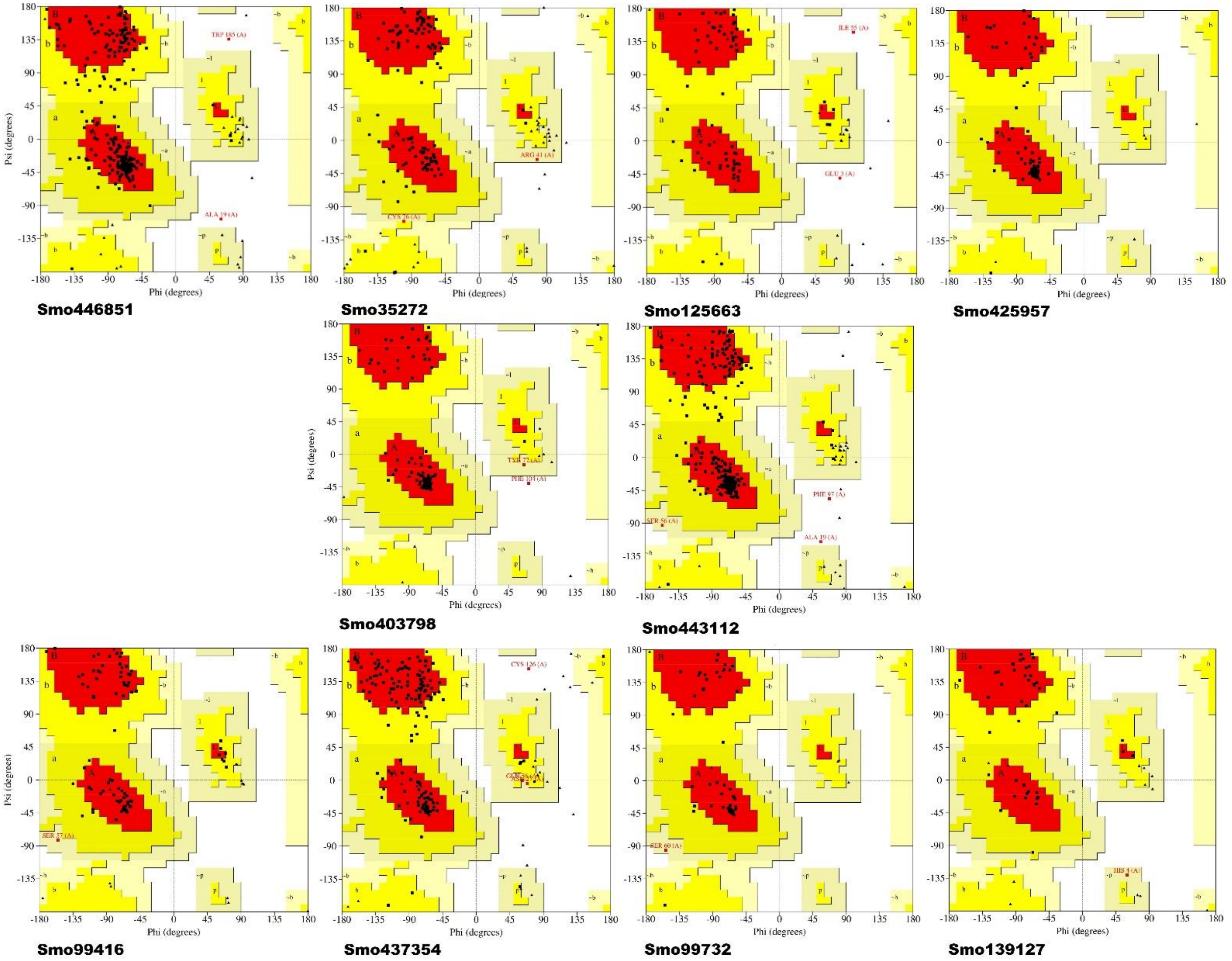

Figure S2: The Ramachandran plots for the Hevein-like lectin model structures.

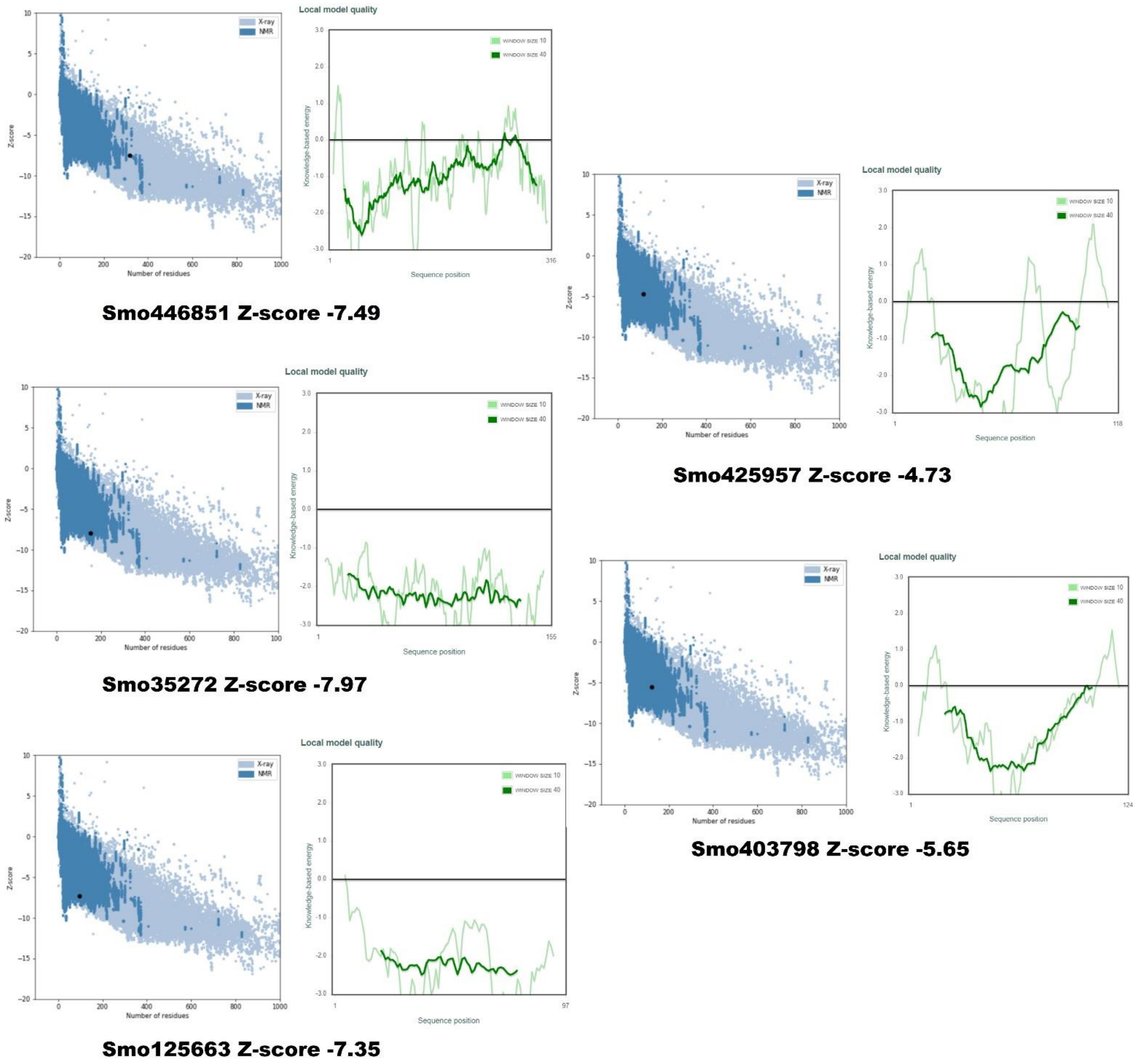

Figure S3-A: The quality and Z-score of the Hevein-like lectin structural models (five models).

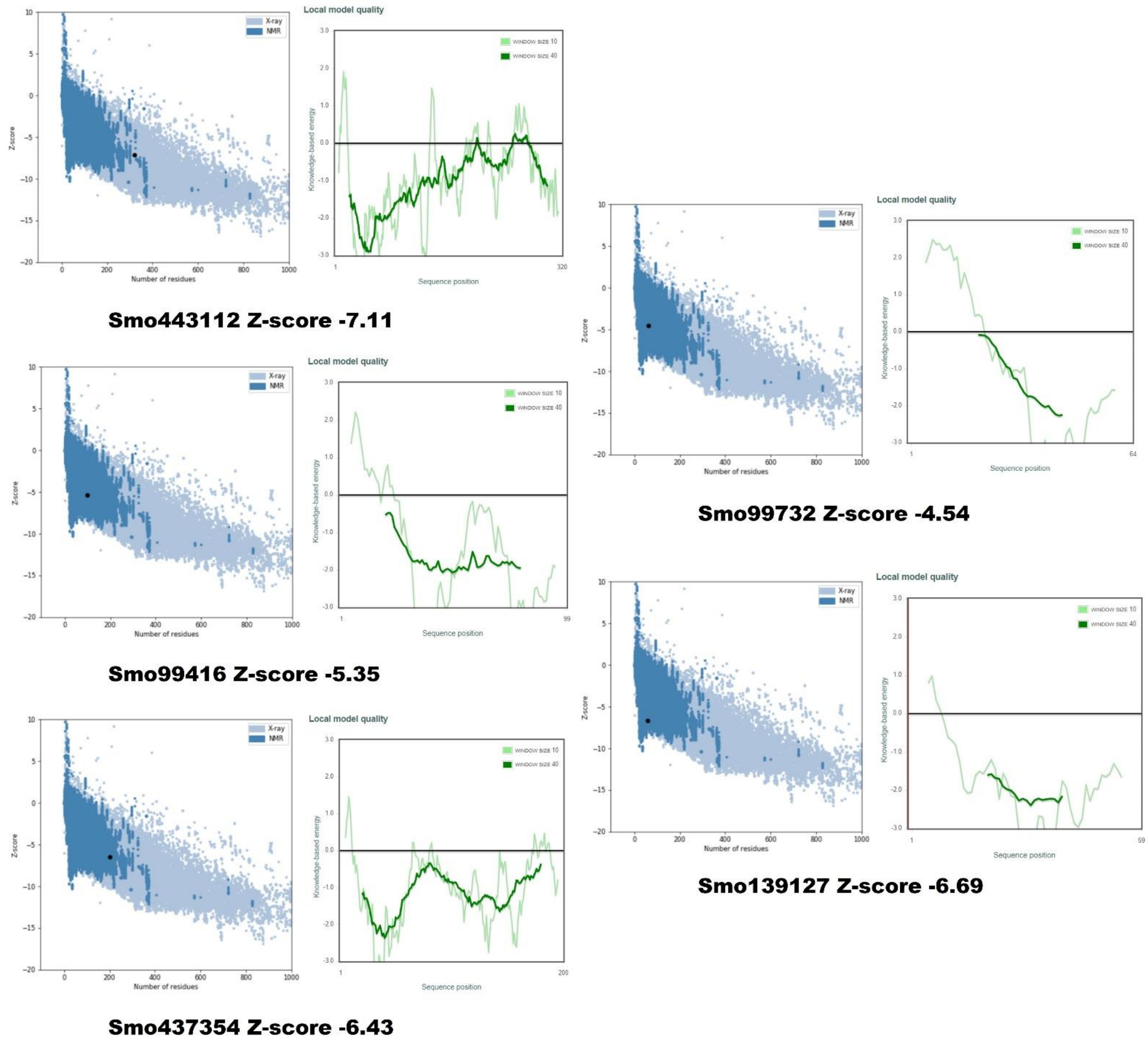

Figure S3-B: The quality and Z-score of the Hevein-like lectin structural models (five models).

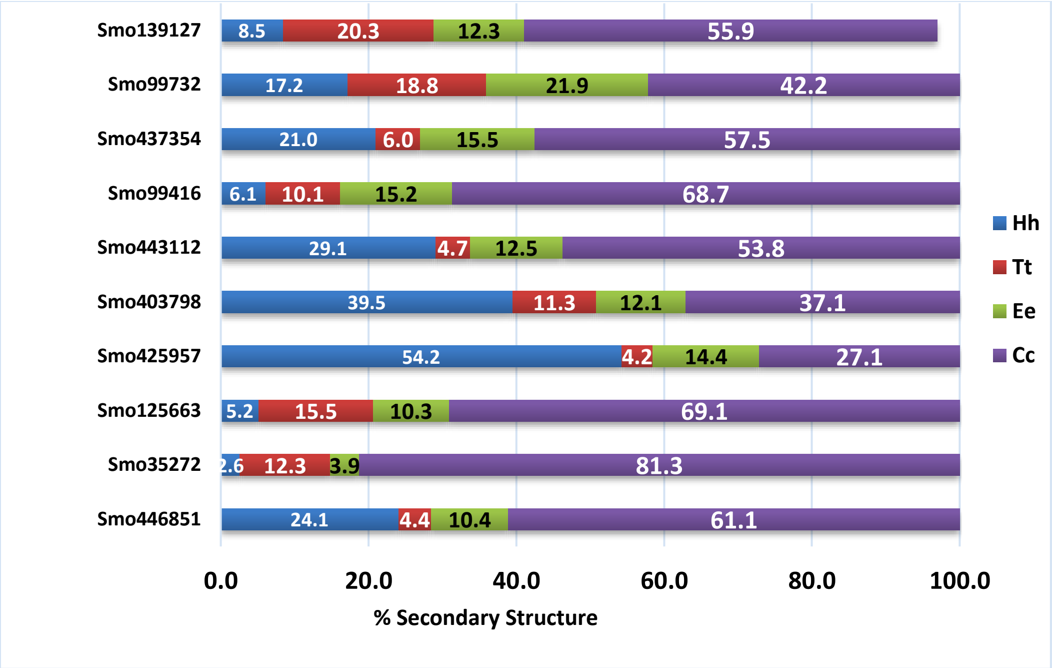

Figure S4: Prediction of the secondary structure of Hevein-like lectins from *Selaginella moellendorffii*. Hh: α-helices, Tt: β-turns, Cc: Random Coil, Ec: Extended strand.

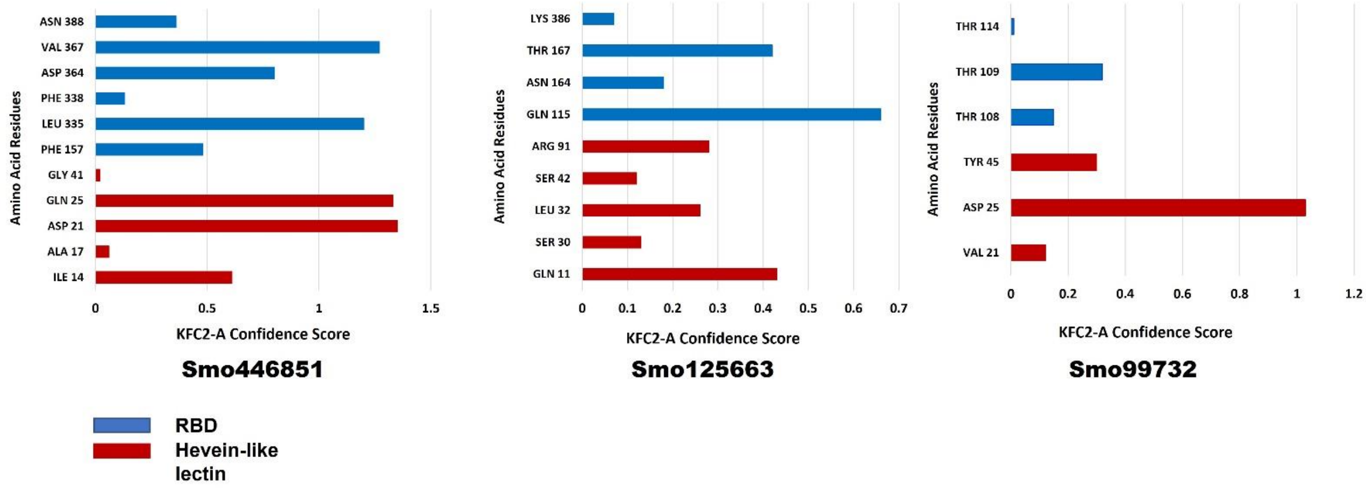

Figure S5: Hot-spot residue scores predicted by KFC-2 for the PPI involving SARS-CoV-2 S glycoprotein.

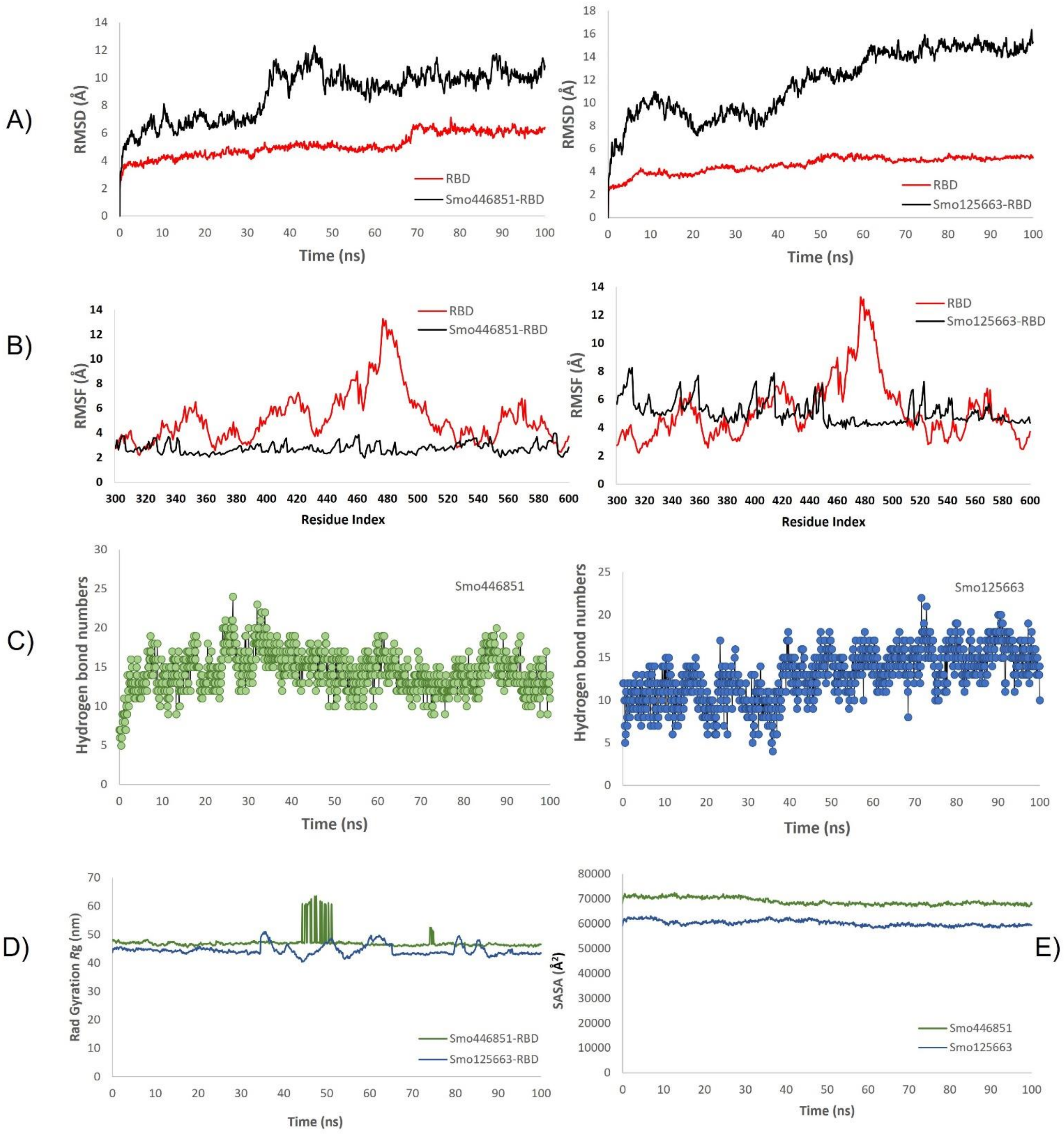

Figure S6: Molecular dynamic simulation (MDS) for the spike protein's RBD complexed with Smo446851 and Smo125663. A) RMSD, B) RMSF, C) Number of hydrogen bonds, D) Radius gyration, E) SASA.

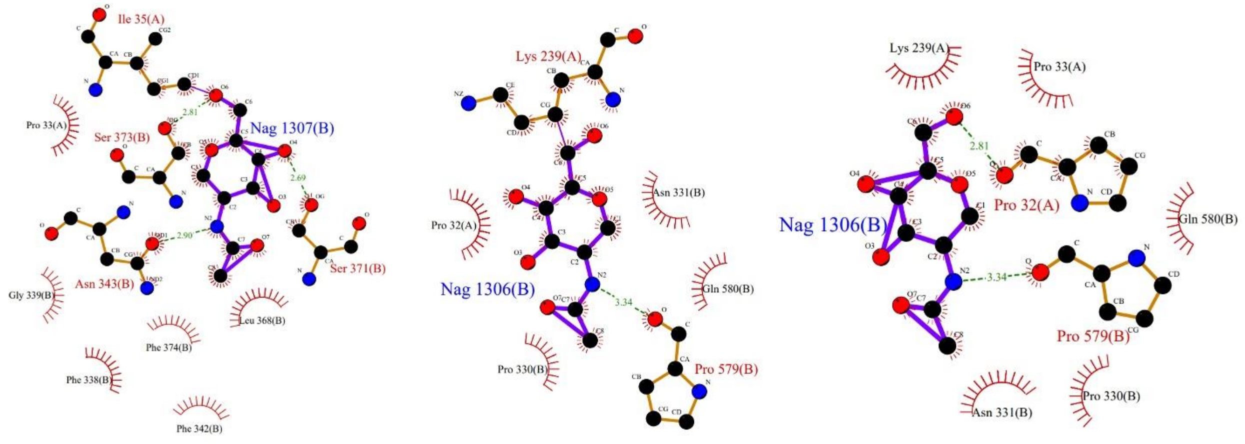

Mutant L452R Mutant T478K Mutant N501Y

Figure S7: NAG1306 and NAG1307 of mutant spike proteins interactions with Smo446851 Hevein domain active residues
